# Brain Function and Upper Limb Outcome in Stroke: A Cross-Sectional fMRI Study

**DOI:** 10.1371/journal.pone.0139746

**Published:** 2015-10-06

**Authors:** Floor E. Buma, Mathijs Raemaekers, Gert Kwakkel, Nick F. Ramsey

**Affiliations:** 1 Centre of Knowledge, Rehabilitation Centre ‘De Hoogstraat’, Utrecht, The Netherlands; 2 Dept. Rehabilitation & Sports Medicine, Brain Center Rudolf Magnus, UMCU, Utrecht, The Netherlands; 3 Dept of Neurology & Neurosurgery, Brain Center Rudolf Magnus, UMCU, Utrecht, The Netherlands; 4 Dept. Rehabilitation Medicine, Research Institute MOVE Amsterdam, VU University Medical Center, Amsterdam, The Netherlands; University of Palermo, ITALY

## Abstract

**Objective:**

The nature of changes in brain activation related to good recovery of arm function after stroke is still unclear. While the notion that this is a reflection of neuronal plasticity has gained much support, confounding by compensatory strategies cannot be ruled out. We address this issue by comparing brain activity in recovered patients 6 months after stroke with healthy controls.

**Methods:**

We included 20 patients with upper limb paresis due to ischemic stroke and 15 controls. We measured brain activation during a finger flexion-extension task with functional MRI, and the relationship between brain activation and hand function. Patients exhibited various levels of recovery, but all were able to perform the task.

**Results:**

Comparison between patients and controls with voxel-wise whole-brain analysis failed to reveal significant differences in brain activation. Equally, a region of interest analysis constrained to the motor network to optimize statistical power, failed to yield any differences. Finally, no significant relationship between brain activation and hand function was found in patients. Patients and controls performed scanner task equally well.

**Conclusion:**

Brain activation and behavioral performance during finger flexion-extensions in (moderately) well recovered patients seems normal. The absence of significant differences in brain activity even in patients with a residual impairment may suggest that infarcts do not necessarily induce reorganization of motor function. While brain activity could be abnormal with higher task demands, this may also introduce performance confounds. It is thus still uncertain to what extent capacity for true neuronal repair after stroke exists.

## Introduction

Stroke is a leading cause of disability in western society [1]. The European Registers of Stroke study (EROS) show that of 2000 patients with first-ever strokes, 40% had a poor outcome in terms of a Barthel Index (BI) below 12 points at 3 months post stroke [2]. In the United States, 50% of stroke survivors suffer from hemiparesis [3,4]. Physical therapy aimed at restoring activities of daily living (ADL) remains the gold standard of treatment but outcomes are variable [5]. Recently, two independent studies have shown that an early return of some shoulder abduction and finger extension within 72 hours post stroke is highly predictive for outcome of upper limb function [6–8]. The patients’ ability to extend the paretic fingers voluntary is seen as an early sign of some intactness of corticospinal tract system (CST) after stroke [7,9]. In addition, in rehabilitation medicine voluntary control of finger extension is judged as a key function for achieving of some dexterity with the paretic limb [6,8,10].

An approach to improve our understanding of the mechanisms underlying functional recovery is to investigate the neural correlates of movement of the affected hand. Many cross-sectional as well as longitudinal studies have previously demonstrated a relationship between various patterns of fMRI brain activation and post-stroke outcome in patients with infarcts that spare M1. Correlations have been found between outcome after stroke, and increased (but also decreased) activation in secondary motor areas (such as PM and SMA), ipsilesional M1 overactivation, contralesional M1 activity as well as more bilateral activation patterns within the motor network, including the cerebellum [11–13]. While there is variation in results of these studies, a recent meta-analysis has shown a consistent pattern of higher contralesional M1 activity and generally more widespread activity in secondary motor areas in stroke patients [14].

The relationship between these changes in brain activation and recovery of motor function is however not necessarily straightforward. Task parameters defining quality of motor performance as well as the occurrence of mirror movements are often not monitored in fMRI and may confound the interpretation of fMRI [12]. In addition, a number of recent longitudinal studies suggest that improvement of upper limb function after stroke is mainly driven by learning compensation strategies rather than by actual neuronal repair [15,16]. In animal studies compensatory strategies as correlates of recovery have also been shown after photothrombotic stroke [17,18]. Patients might learn to deal with impairments by using the affected limb to perform a task in a different way than before the stroke using alternative neuronal pathways, for example by reducing the number of degrees of freedom during movement [16,19–21]. While such strategies may underlie clinical improvement, they do not constitute true neuronal plasticity or repair.

In the present fMRI study brain activity during motor function while performing an isolated, voluntary finger extension motor paradigm, is compared between patients with damage to the corticospinal tract and healthy controls. The patients are measured >6 months after stroke, when most of the recovery would be expected to have taken place. In addition, the quality of task performance was closely monitored with kinematic measurements to detect potential performance confounds, so that observed differences in brain activation between patients and control subjects can potentially be directly linked to neuronal plasticity [12]. We hypothesize that extent of functional recovery after stroke is associated with reorganization of brain function during a motor task, as proposed in literature [9,22]. We expect task-related brain activation to differ between subjects that have shown some motor recovery of the upper paretic limb, and healthy, age-matched controls. Specifically, we expect to find in stroke patients 1) elevated activation of secondary motor areas, 2) a more bilateral activation pattern across the motor network, as well as 3) a correlation between brain activity and functional outcome. However, we observed that under these well controlled conditions, there were differences in brain activation between patients and control subjects.

## Materials and Methods

### Subjects

Twenty patients with chronic stroke and fifteen healthy, age matched controls were included. All patients were measured at least 5 months after a first-ever ischemic stroke, at which time point most of the functional recovery has already occurred [23]. Patients had no previous history of other neurological conditions. Clinical characteristics of patients studied are described in Table 1. Patients had a mean age of 56 years and 5 months (SD 10 years 4 months) and control subjects mean age was 55 years 11 months (SD 9 years 1 month). Groups were matched on age, sex and dexterity. Patients were included if, in the first weeks after stroke they had suffered from hemiparesis or paralysis of the hand. Further patient inclusion criteria were: age between 18 and 80 years, ability to understand instructions (score above 22 on the mini mental state examination (MMSE)) [24]. Exclusion criteria consisted of: orthopedic restrictions of the upper extremities; botulin toxin injections or other medication influencing the function of the upper extremity. Subjects gave written informed consent. The protocol was approved by the ethical board of the University Medical Center Utrecht, and was in accordance with the Declaration of Helsinki (2008).

**Table 1:** Patient Characteristics.

Patient	Age(Years)	TPS(Months)	Gender	Hand	Hem	Location	FM	ARAT	%NHPT*
**1**	42	26	F	L	L	SC	66	57	86
**2**	52	24	M	R	R	P	61	57	80
**3**	53	46	F	R	L	C	61	57	76
**4**	47	45	M	R	R	SC	56	52	72
**5**	67	31	M	R	L	SC	63	57	62
**6**	67	33	M	R	R	SC	53	56	65
**7**	73	22	M	R	R	P	66	57	125
**8**	57	36	M	R	L	C	58	57	65
**9**	57	41	M	A	R	SC	65	57	84
**10**	60	14	M	NA	R	SC	66	57	67
**11**	50	5	M	R	R	SC	59	57	69
**12**	73	22	M	R	L	C	44	50	57
**13**	48	39	M	R	L	SC	57	53	18
**14**	73	113	M	R+	R	SC	66	57	100
**15**	49	26	M	R	L	SC	55	57	58
**16**	40	128	F	L	R	SC	64	57	46
**17**	64	20	M	R+	L	SC	61	57	82
**18**	59	21	F	R	L	SC	61	53	34
**19**	45	11	M	R	L	P	61	57	70
**20**	53	14	F	R	R	SC	66	57	65
**Mean**	56.5±10.3	35.9±31.0	5 F/15 M	2L/14R/2R+/1 A	10L/10R	3P/3C/14SC	60.5±5.6	56.0±2.1	69.25±22.55
**Mean controls**	55.9±9.1		5F/10M	1L/13R/1A					

Abbreviations: TPS time post stroke, M Male, F Female, Hand Handedness (Dexterity was established by the Edinburgh Hand Inventory), R right, L left, R+ forced to write, A ambidextrous, Hem lesioned hemisphere, P pontine, C extending to cortex, SC subcortical.

*NHPT results are given as percentage of norm scores (corrected for age and handedness).

### Clinical Assessments

Motor function of the affected arm of each patient was rated using the upper extremity motor part of the Fugl-Meyer (FM-arm) test, the Action Research Arm Test (ARAT) and the nine-hole peg test (NHPT) at the time of fMRI measurement. The FM-arm is a test based on the concept of sequential stages of return of motor function [25] and it tests reflexes, synergy of the upper extremities as well as hand function. The assessments are scored on an ordinal 3-point scale to express a maximum motor score for the affected side, with a total score ranging from 0 to 66. The ARAT is a quantitative test of arm motor function [26]. Hand movements, including pinch, grasp, grip and gross, are performed and scored on a 4-point scale, with a total score ranging from 0 to 57. The ARAT score can be divided into 3 categories, poor, moderate or good recovery (i.e. <10 points, 10–56 points, or 57 points) [27]. The NHPT measures dexterity of the hand, focusing on fine motor function. Pegs are inserted and removed from a nine-hole peg-board. Scores are based on the time (in seconds) taken to complete the test and are calculated as a percentage of a healthy sample norm adjusted for age, sex, and handedness [28,29].

### Data Acquisition

Images were acquired with a Philips Achieva 3.0 Tesla MR scanner (Philips Healthcare, Eindhoven, Netherlands). A 3D PRESTO sequence was used for functional scanning (FA = 10 degrees, FOV = 224 × 256 × 160 mm, voxel size 4 × 4 × 4 mm, TE/TR = 33/23 ms, time per 40-slice whole-brain volume 0.63 s) [30]. High-resolution whole brain anatomical scans were acquired for all subjects as reference for functional activation maps (3D T1-weighted scan: TR = 9.717 ms; TE = 4.59 ms, flip angle = 8 degrees, 140 slices, 0.875 x 0.857 x 1.2 mm, FOV = 224 x168 x177 mm). Electromyography (EMG) was measured during scanning over the extensor digitorum communis of the hand contralateral to the moving hand with four scanner compatible surface electrodes. The EMG electrodes were attached to the connector on the scanner for physiological synchronization. The EMG was acquired to detect and control for isometric contractions of the hand contralateral to the hand that was instructed to move [31]. In addition, two MR-compatible data gloves (5DT Inc.) were used to measure overt hand movements [32].

### Motor Paradigm

Patients were asked to perform two different motor tasks in the MRI scanner, consisting of flexion and extension of the fingers of the hand (alternating 20 seconds of movement and 20 seconds of rest for a period of 6 minutes per task).

Before fMRI scanning, subjects were trained to perform active extension movements with the fingers, using a plastic wrist-hand orthosis. The orthosis guaranteed a correct movement in the flexion–extension direction. To maximize mental engagement during the task, the active extension of the fingers varied in amplitude of movement for the first task, and varied in exerted force during extension for the second task. The two tasks used similar visual stimuli. For the first task (AMP), subjects wore a data glove on each hand, and movement amplitude was varied by subjects themselves while they were guided by an online visual representation of their movement, as assessed with the data glove of the hand that was instructed to moves. Both arms rested comfortably in a supine position supported by cushions next to the patients hips, with the elbows slightly bent in a comfortable position for each patient. The average position of the fingers was calculated based on the average angle between the extended fingers and the hand. The signal was calibrated by asking the subject to bend the stretched fingers in a 90 degree angle, and then stretch the fingers in line with the hand. The calibration was visually inspected by a researcher who was present in the scanner room at all times. The task was presented on a screen, with graphical instructions. On the left, the target cue moved vertically moving up (representing stretching of the fingers) and down (representing bending the fingers in 90 degrees flexion). On the right side of the screen feedback was given (as an object also moving up and down a vertical line) of the actual position of the hand through online processing of the signals from the data glove. Subjects were asked to make the feedback object follow the target cue to the best of their ability. A movement cycle of the cue lasted 1 second and changed color to inform the patient that a rest or a move block was indicated. The requested amplitude of finger extension was varied between blocks at 3 levels (low, medium and full extension). The height of the target cue indicated the level of finger extension.

For the second task (FORCE) the requested force for the movement was varied between blocks by attaching 0, 1 or 2 elastic bands to the orthosis. The requested amplitude of the movement during the force task was at maximum (between 0 and 90 degrees), as guided by the visual cue. The amount of required force was thus kept the same for all subjects. No data-glove measurements were obtained during the FORCE task, as the orthosis that was used introduced physical constraints so that it could not be combined with the data glove. All subjects performed both tasks with the affected as well as the unaffected hand or right and left hand in controls, making a total of four tasks per subject. Visual inspection by a researcher who was in the scanner room during scanning, confirmed that all patients extended their fingers maximally in response to the changing force.

### Data Preprocessing fMRI

All spatial preprocessing and first level analyses were done with statistical Parametric Mapping (SPM5) software (http://www.fil.ion.ucl.ac.uk/spm/) running in MATLAB (Mathworks Inc, Massachusetts, USA). All functional images of each participant were realigned to the first scan of each session, using 5 mm FWHM spatial smoothing during parameter estimation. After realignment, all imaging data were coregistered to the T1-weighted anatomical scan using a mutual information cost-function with 7 x 7 pixels FWHM histogram smoothing. Subsequently, images were normalized to the Montreal Neurological Institute brain using the unified segmentation procedure of SPM, which can perform intersubject image registration based on tissue classification maps [33]. To prevent incorrect warping near the lesions, the ischemic lesions were masked during the segmentation. The masks were generated by manually drawing borders around the lesion in MRIcro (http:/www.psychology.nottingham.ac.uk/staff/cr1/mricro.html), and subsequently inverted so that voxels in and around the area affected by stroke could not contribute to the establishment of the normalization parameters. Motion-related and high frequency artifacts were removed from the normalized timeseries data using MELODIC of the FMRIB software library [34]. in combination with a General Linear Model (GLM).

The resulting normalized images were spatially smoothed for voxelwise group comparisons using a Gaussian filter of 8-mm full width at half maximum. Unsmoothed data were kept for an ROI analysis. The design matrix for the first level analysis was generated, using a high-pass filter with a cutoff at 128 seconds to remove low-frequency artifacts and correction for serial correlations with an autoregressive model.

Contrast maps were calculated for the active periods versus rest for each subject and each session. Contrast images from ten patients with right-sided lesions were flipped over the mid-sagittal plane, so that the affected hemisphere corresponded to the left side of the brain for all patients. The same was done with 7 matched controls to match groups.

### Groupwise Comparisons of fMRI Data

**Fig 1:**
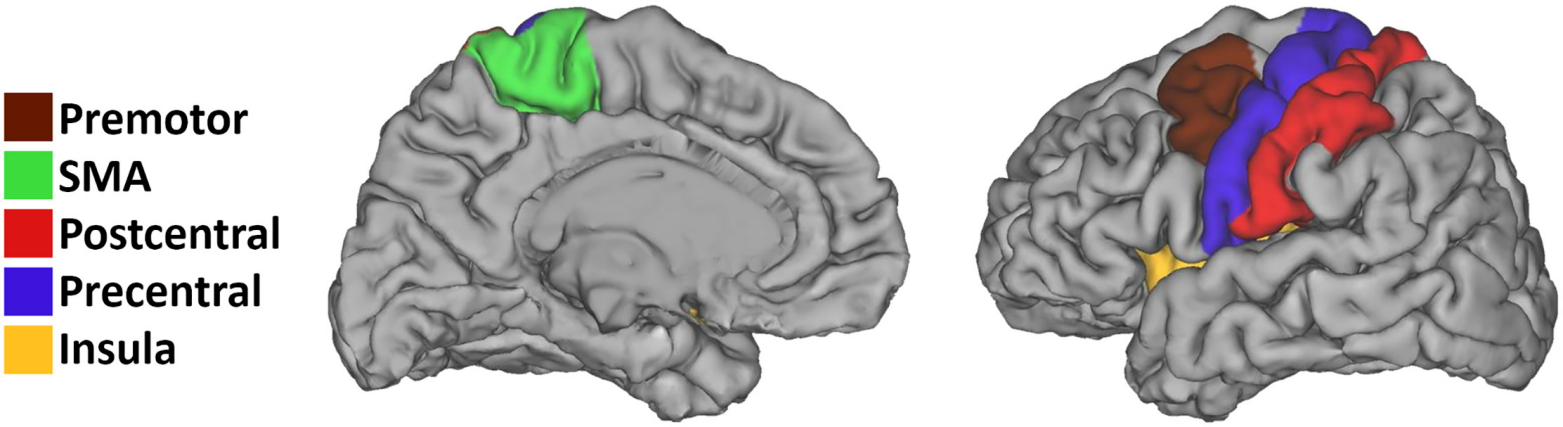
Surface reconstruction of a single subject with the anatomical motor segments depicted by different colors.

An ROI based comparison was performed using the unsmoothed fMRI data. ROI's were generated by an automatic segmentation that was applied to all subjects anatomical image to delineate the cortical areas using Freesurfer [35]. This automatic delineation is performed on the basis of geometric information of individual cortical model as well as neuroanatomical convention, and does not require explicit back-projection from a template segmentation to generate ROI's. The motor segments were selected from the segmentation and ROI’s were generated by taking the 15% most active voxels (i.e. highest beta values within a segment) during the motor task (task vs. rest) in each anatomical motor segment (Supplementary Motor Area (SMA), Premotor area (PM), precentral and postcentral gyrus, insula and cerebellum; see Fig 1 for an example).

A proportional instead of absolute threshold was used in the ROI definition to account for global signal variations [36]. The choice of 15% was based on a rough estimate of the mean volume of activation across the included ROI's, which was based on data of previous work of our group regarding reliability of fMRI motor activation [37]. BOLD signal changes per ROI were represented by the mean beta value during each task. All the motor segments were then visually inspected to ensure correct segmentation for each subject. All selected motor segments were unaffected by the lesion, which were mainly subcortical or in some cases extending to other cortical areas (Fig 1). The segmentation maps were normalized to MNI space with the previously estimated normalization parameters. ROI's included bilateral precentral and postcentral gyrus, SMA, PM, and cerebellum.

In addition a laterality index (LI) was determined for the different motor areas by selecting the top 15% voxels in the bilateral anatomical motor segments (combined left and right), and counting the number of voxels selected in each segment. The laterality index was defined as LI = (vox_i_−vox_c_)/ (vox_i_ + vox_c_), where vox_c_ and vox_i_ denote the number of voxels of the hemisphere contralateral and ipsilateral to the lesion respectively [22]. The LI ranges from 1 (all activated voxels are in the ipsilesional hemisphere) to -1 (all activated voxels are in the contralesional hemisphere). Differences in the activation in the ROI’s between patients and controls were tested with a general linear model (repeated measures ANOVA) with ROI (6 levels), hemisphere (2 levels) and amount of force/amplitude (3 levels) as within-subjects factors. In addition to the ROI based analysis, a voxelwise group analysis was performed in MNI template space to test for possible differences outside the predefined ROIs. Voxelwise differences in the activation maps between groups were estimated with an independent samples t-test in SPM5. The resulting statistical maps were thresholded at p <0.05 (corrected) [38].

### Correlation with Outcome

To assess whether task related activity in the ROI’s was predictive of outcome, a design matrix was constructed for each task, with each factor in the design matrix representing the activation in a single ROI for each patients. The three design matrices were applied to each of the behavioral measures (%NHPT, FM, and ARAT scores) in a stepwise regression procedure. The threshold for inclusion of factors in the model was set at p<0.05, and at p>0.10 for exclusion.

### Data Glove and EMG Analysis

The signal of the data-glove and EMG data were analyzed offline with MATLAB. The signal from the data-glove was high-pass filtered to correct for drift in the signal and resampled to a 15 ms temporal resolution. Subsequently, the number of hand movements was derived by counting the number of maxima and minima of the movement signal and then dividing that number by two. The correlation coefficient of the envelope of the movement signal with the task boxcar was calculated to assess the adherence to the changing amplitude and timing of the task.

The EMG signal was analysed using a previous established approach [39,40]. To remove fMRI artefacts induced by the gradient magnets, the EMG signal was notch filtered at 45 and 90 Hz. Second the signal was high pass filtered at 10 Hz to remove movement artefacts. Third the signal was rectified to regain low frequency components, the signal was rectified. Data were then band-pass filtered between 2 and 130 Hz and a correlation coefficient was calculated for the envelope of the signal time series and the task as a boxcar function.

Subjects were asked to perform a maximal voluntary extension (MVE) of the fingers before every task in the scanner. The corresponding EMG signal over that time was averaged and used as a norm value for average %MVE ($\bar{\%MVE}$) during movement blocks. $\bar{\%MVE}$ was calculated by dividing the average EMG signal during the task by the average MVE and multiplying this by 100%.

$$\bar{\%MVE}=\frac{\bar{\text{EMG}}}{\text{MVE}}\cdot 100$$

This scaling was performed to account for intersubject variation in the amplitude of the signal as a result of factors such as conductivity of the skin, amount of muscle tissue, and the exact locations of the electrodes on the hand. EMG Mirror Movements (MM_EMG_) were represented by the correlation coefficient of the envelope of the EMG signal (E_EMG_) and the task boxcar (T) multiplied with the $\;\bar{\%MVE}$.

$$MM_{EMG}=r_{T,E_{EMG}}\cdot \bar{\%MVE}$$

## Results

### Clinical Data

**Fig 2:**
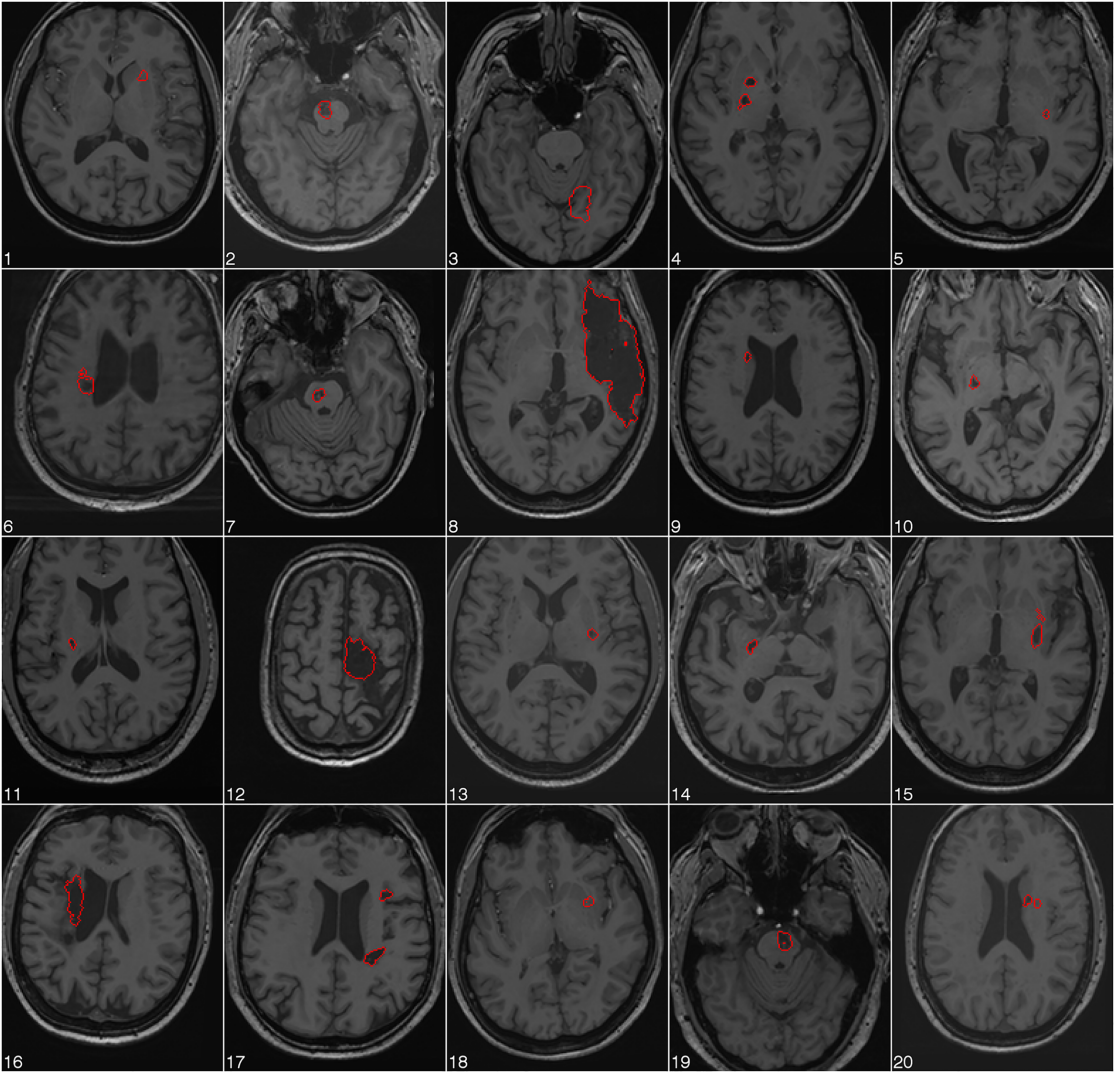
Axial structural T1-weighted MRI scans at the level of maximum infarct volume for each patient performed at the time of the fMRI session.

The site of cerebral infarction was determined from the structural MR images (Fig 2). Fourteen patients had subcortical infarctions in the capsular region, 3 patients had pontine infarctions, and 3 patients had infarctions extending into the cortex. No infarcts included motor cortex (Brodmann area 4). At the time of the measurement patients were on average at 36 months (SD = 31 months) post stroke.

On the ARAT test (Table 1) patients scored significantly less than the maximum score of 57 at an average of 56 (one-sample Wilcoxon rank test: p = 0.042). On the FM-arm test (Table 1) patients scores 60 points on average, which is significantly lower than the maximum score of 66 points (one-sample Wilcoxon rank test: p<0.001). On the NHPT patients scored a mean of 69.3% of norm values, which is significantly lower than %100 (one-sample T-test: p<0.001). Hence, as a group the patients were not fully recovered.

### Glove Data

Problems with the acquisition hardware resulted in the absence of glove data for a total of 6 tasks in 5 subjects. All patients were able to perform the flexion/extension (AMP) task during scanning. A 2-sample t-test showed no difference for either amplitude or frequency of movements during the amplitude task between patients and controls for both hands (t_33_<1; p>0.4 for all tests). In addition no actual mirror movements were seen in patients as well as controls during the amplitude task, as shown by a low correlation (MM_glove_) of the inactive hand with the task (mean r = -0.02 for the unaffected arm; mean r = 0.00 for the affected arm). A paired t-test also showed no difference in amplitude or frequency of movements between the affected and unaffected hand movements in patients as well as right and left hand movements in controls(t_18_<1; p>0.401 for patients; t_13_<1.538; p>0.14 for controls. For individual data, see Table 2.

**Table 2:** Results from analysis of data-glove data on task performance and mirror movements and scores on isometric contractions derived from EMG-data for patients.

P	MM_EMG_ score				MM_glove_		Compliance Correlation		Number of movements (Hz)	
	UA	AA	UF	AF	UA	AA	UA	AA	UA	AA
**1**	0.43	0.08	0.07	0.59	-0.17	-0.16	0.77	0.78	92	89
**2**	0.64	0.77	0.22	0.01	-0.08	0.16	0.82	0.79	92	92
**3**	0.02	0.06	0.00	0.26	-0.12	-0.16	0.89	0.88	92	89
**4**	0.00	0.78	0.04	0.08	-0.03	-0.04	0.68	0.77	98	98
**5**	NA	0.60	0.02	0.02	-0.17	NA	0.96	NA	87	NA
**6**	0.07	1.13	0.01	0.01	0.33	-0.15	0.39	0.47	81	84
**7**	4.65	10.80	4.95	43.18	NA	NA	NA	NA	NA	NA
**8**	NA	NA	20.13	NA	0.06	-0.03	0.27	0.83	86	86
**9**	0.16	0.02	0.01	4.10	0.19	-0.01	0.71	0.6	75	64
**10**	0.07	0.00	0.19	0.44	0.15	-0.06	0.8	0.8	91	95
**11**	2.40	24.50	0.05	1.06	-0.09	-0.13	0.85	0.52	90	84
**12**	0.05	0.27	0.07	0.35	-0.12	-0.15	0.87	0.66	88	89
**13**	0.01	9.02	0.01	45.13	-0.18	0.38	0.86	0.85	97	92
**14**	0.01	1.38	0.02	0.15	0.01	NA	0.15	NA	85	NA
**15**	4.55	0.08	0.05	0.97	-0.07	0.01	0.61	0.78	86	93
**16**	1.48	0.77	0.00	0.61	0.08	-0.09	0.67	0.48	102	86
**17**	0.31	0.11	0.13	0.75	-0.03	-0.07	0.72	0.66	98	98
**18**	0.33	0.25	1.35	0.22	-0.04	0.05	0.77	0.68	95	93
**19**	0.08	0.21	0.05	0.30	-0.04	0.21	0.86	0.85	94	82
**20**	0.03	0.08	25.27	0.03	-0.03	0.16	0.72	0.55	90	94
**Mean**	0.85	2.68	2.63	5.17	-0.02	0.00	0.70	0.70	89.95	88.71
**SD**	1.50	6.09	7.00	13.77	0.13	0.15	0.22	0.14	5.83	7.96
**Independent t-test, differences patients and controls**										
**t-test**	.808	.912	.389	.861			-.291	.796	-.499	-1.14
**P**	.426	.545	.700	.401			.77	.62	.62	.26
**Paired t-test, Affected vs. unaffected hand**										
**t-test**	.712		.861				.390		1.538	
**P**	.488		.401				.70		.14	

Abbreviations: EMG Electromyography, MM mirror movements, SD standard deviation, t-test student’s t test statistic, p p-value for student’s t test statistic, UA unaffected amplitude, AA affected amplitude, UF unaffected force, AF affected force, NA Data unavailable (due to malfunction of equipment), $\bar{\%MVE}$ percentage of EMG signal during maximum voluntary contraction.

### EMG Data

**Fig 3:**
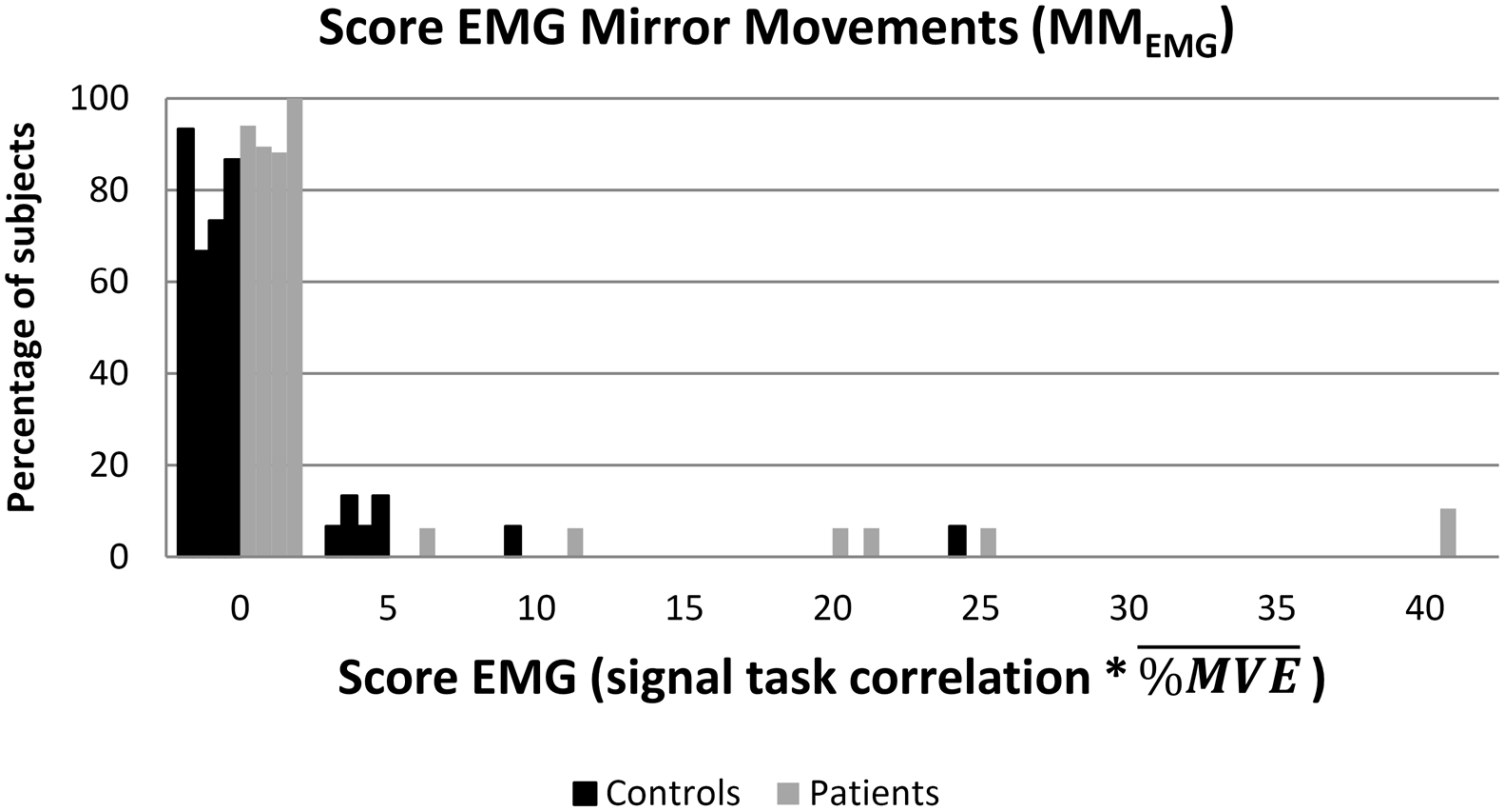
Histogram of incidence of isometric contractions (MM) in the contralateral hand defined by a score consisting of the correlation of the electromyography (EMG) signal measured at the extensor muscles of the contralateral hand during the task with the task boxcar multiplied by the average % of maximal voluntary contraction ($\bar{\%MVE}$) (measured before the session in the scanner) of the muscle during the session. Patient-scores are depicted in black. Control-scores are depicted in grey.

Problems with the acquisition hardware resulted in the absence of EMG data for 3 tasks in 1 subject. No difference was found in EMG activity scores between patients and controls, or between affected and unaffected hand movements for patients, or between left and right hand movements for controls (Fig 3). A number of patients as well as controls had a high score (Score ≥5) for the EMG data during some sessions. The reason for these high scores remains elusive. However, a high EMG score during one session did not automatically mean a high score during other sessions, or during movements of the other hand, meaning that a relationship with stroke is unlikely (for individual data see Tables 2 and 3).

**Table 3:** Results from analysis of data-glove data on task performance and mirror movements and scores on isometric contractions derived from EMG-data for controls.

C	MM_EMG_ score				MM_glove_		Compliance Correlation		Hz	
	UA	AA	UF	AF	UA	AA	UA	AA	UA	AA
**1**	0.18	0.16	0.36	0.20	0.02	0	0.71	0.71	89	89
**2**	4.47	2.09	0.39	0.33	0.01	-0.06	0.91	0.87	97	92
**3**	3.33	1.57	1.63	0.00	0.06	-0.27	0.19	0.27	81	96
**4**	0.39	0.86	0.02	0.01	0	-0.01	0.81	0.51	93	100
**5**	0.05	0.23	0.29	0.01	-0.15	NA	0.9	NA	90	NA
**6**	0.01	0.02	2.57	0.15	-0.11	-0.2	0.35	0.7	93	97
**7**	0.01	0.09	0.11	4.54	0.03	0.06	0.92	0.88	92	93
**8**	0.08	6.01	0.03	0.00	0.18	0.1	0.83	0.76	90	94
**9**	3.42	0.03	0.66	3.75	0.07	NA	0.86	NA	93	NA
**10**	0.23	0.54	2.87	4.64	0.11	-0.17	0.63	0.49	95	93
**11**	1.30	10.39	1.23	9.84	0.19	-0.2	0.56	0.81	82	88
**12**	7.89	0.46	1.17	1.06	-0.1	-0.32	0.72	0.62	94	93
**13**	0.08	0.99	5.96	0.00	0.36	0.01	0.74	0.43	105	80
**14**	0.03	0.00	0.04	0.26	-0.01	-0.07	0.88	0.82	91	85
**15**	0.34	29.91	0.02	7.36	-0.06	-0.06	0.87	0.66	88	91
**Mean**	1.45	3.56	1.16	2.14	0.04	-0.09	0.73	0.66	91.53	91.62
**SD**	2.32	7.83	1.62	3.16	0.13	0.13	0.22	0.19	5.74	5.24
**Paired t-test. Affected vs. unaffected hand**										
**t-test**	-.394		-.525				.858		-.030	
**P**	.699		.611				0.41		0.98	

Abbreviations: EMG Electromyography, MM mirror movements, SD standard deviation, t-test student’s t test statistic, p p-value for student’s t test statistic, UA unaffected amplitude, AA affected amplitude, UF unaffected force, AF affected force, NA Data unavailable (due to malfunction of equipment), $\bar{\%MVE}$ % of EMG signal during maximum voluntary contraction.

Since no actual movements were detected in the contralateral hand with the data-glove, a high score in EMG data seen in some subjects is more likely a representation of isometric contractions of the hand extensor muscles, and not a representation of EMG signal correlating with actual movement.

### Imaging Results

**Fig 4:**
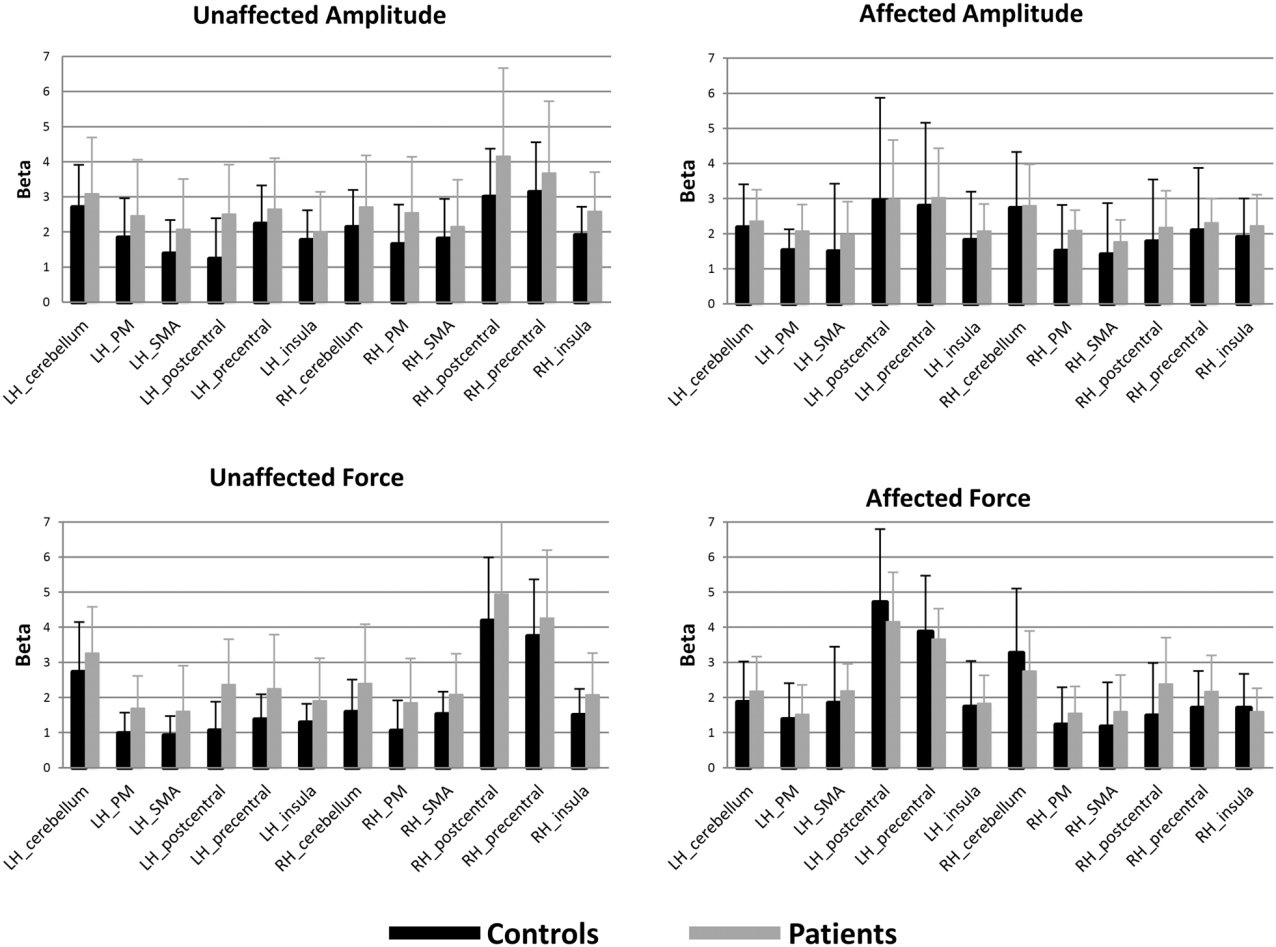
Mean results for Amplitude and Force tasks for the unaffected and affected hand for patients and controls. Bars show the mean beta per ROI (±1 SD) cerebellum, PM, SMA, postcentral gyrus, precentral gyrus and insula for the left (affected) and right (unaffected) hemisphere (LH, RH). Patients’ T-maps were flipped so affected hand was always the right hand.

The activation levels for all conditions in the different ROIs for each subject can be seen in the spreadsheet in S1 File. ROI analysis for Cerebellum, SMA, PM, precentral cortex, postcentral cortex and insula did not show differences between groups for affected amplitude, affected force, unaffected amplitude, or unaffected force (Table 4, Fig 4).

**Table 4:** Results ANOVA Differences in brain activation between and within groups.

Contrast	Betas	LI
**Patients vs. Controls**		
**Affected Amplitude**	F = 0.333;p = 0.568 ^1^	F = 0.439;p = 0.512 ^2^
**Affected Force**	F = 2.422;p = 0.129 ^1^	F = 0.225;p = 0.638 ^2^
**Unaffected Amplitude**	F = 1.028;p = 0.318 ^1^	F = 1.774;p = 0.192 ^2^
**Unaffected Force**	F = 0.540;p = 0.468 ^1^	F = 2.077;p = 0.159 ^2^
**Affected vs. Unaffected hand**		
**Force Patients**	F = 0.733;p = 0.403 ^3^	F = 0.422;p = 0.524 ^4^
**Force Controls**	F = 0.397;p = 0.539 ^3^	F = 1.066;p = 0.319 ^4^
**Amplitude Patients**	F = 1.642;p = 0.215 ^3^	F = 1.259;p = 0.276 ^4^
**Amplitude Controls**	F = 0.916;p = 0.355 ^3^	F = 0.600;p = 0.452 ^4^

Abbreviations: F value for F-statistic, p p-value for f-statistic.

^1^ = group * ROI * hemisphere interaction

^2^ = group * ROI interaction

^3^ = condition * ROI * hemisphere interaction

^4^ = condition * ROI interaction

**Fig 5:**
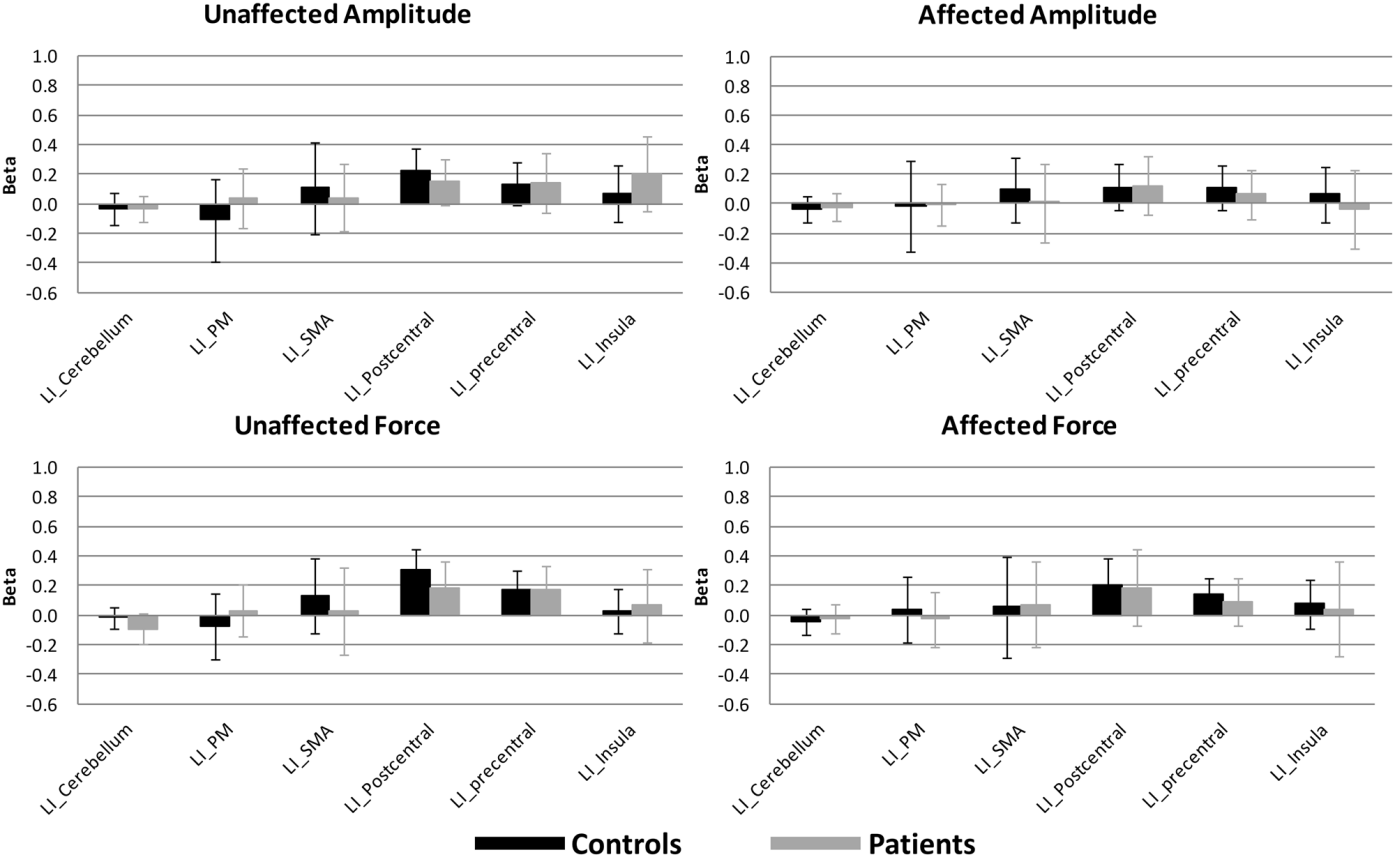
Mean results for Laterality Index for Amplitude and Force tasks for the unaffected and affected hand for patients and controls. Mean LI per ROI (±1 SD) cerebellum, PM, SMA, postcentral gyrus, precentral gyrus and insula.

In addition, there was no difference in ipsi- or contralesional ROI activity between the affected and unaffected hand neither for patients nor for controls. The laterality index did not show a significant effect for group (Table 4, Fig 5), and did not show a difference between affected and unaffected hands for patients or for controls. There was no interaction effect for group with task, ROI, or hemisphere. To see if significant results were absent due to heterogeneity in lesion location, we repeated the ROI analysis with inclusion of only patients with lesions in the basal ganglia, the largest subgroup. However, still none of the tasks showed a significant effect regarding activity levels (betas) or laterality indices. In addition, we repeated the analysis within patients (affected vs. unaffected) while including time post stroke as covariate, to nullify potential within group variance as a result of different levels of functional reorganization as a consequence of between subject differences in time post stroke. Again this did not produce significant effects for any of the tasks.

**Fig 6:**
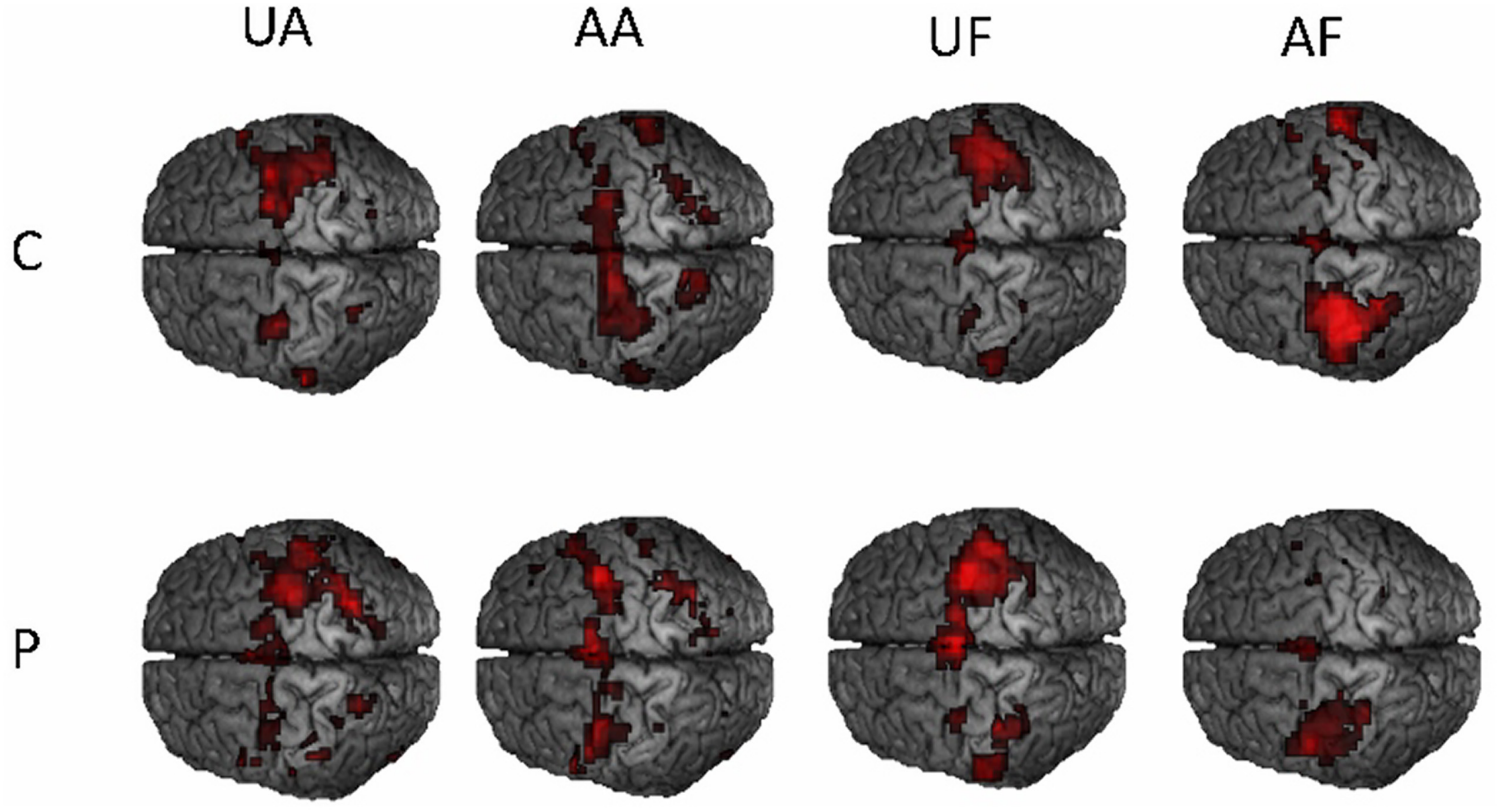
Group activation move vs. rest for 4 sessions, unaffected amplitude (UA), affected amplitude (AA), unaffected force (UF), and affected force (AF) between patients (P) and controls(C). For illustration purposes threshold was set at T>5 uncorrected.

Voxelwise group comparisons were made with SPM for each task separately, for both groups separately and for the difference between patients and control subjects. The contrast maps in niftii format for each patient and control subject and for each condition can be found in a ZIP file archive in the supporting files (S2 File for patients and S3 File for control subjects). The analysis of the main effect (flexion-extension compared with rest) of the amplitude as well as the force task revealed activation in a broad network of brain regions (Fig 6). The most lateralized activation was in the sensorimotor cortex and superior cerebellum, with larger activation contralateral and ipsilateral to the moved hand respectively. Other activations were more bilateral, including the PM, SMA, inferior parietal cortex and, insular cortex, and bilateral cerebellum. Comparison between patients and controls did not reveal any significant increase or decrease in activation for the amplitude or force task and for either hand. Comparison between affected and unaffected hand movements also did not reveal any difference in activation for patients, nor for controls.

During stepwise regression none of the factors of the design matrices were included. This means that there was no significant correlation in any of the ROI’s between task-related activity and %NHPT, ARAT or FM scores. In addition, LI scores did not correlate with clinical assessments for any of the sessions. There was no effect of mirror movements assessed with either EMG measures or data glove on brain activity. EMG and glove mirror movement scores did not correlate with task-related activity in any of the ROI’s.

## Discussion

Patients exhibited various degrees of recovery with mainly reduced scores on the NHPT and less so on the FM. While significantly lower than the maximum score, there was no clinically meaningful reduction (>6 points) in average scores for the ARAT, indicating good performance in general [41]. Brain activation during movement of the affected hand was not different for patients and controls with either a voxelwise whole brain analysis in MNI template space, or statistically more powerful ROI analyses including regions of the motor system. Moreover, the data showed no significant correlation between brain activation during any of the flexion-extension tasks, and functional outcome measures, in spite of the fairly wide range in outcome on the NHPT (%NHPT 18–125%). Detailed monitoring of movement extent and rate, and of mirror movements, indicated that patients and controls did not differ significantly with regard to any of these measures. We thus could not confirm an association between partial or complete recovery from stroke affecting the upper limbs and altered engagement of secondary or more bilateral motor areas, even when residual impairment is evident as demonstrated with the NHPT. The data thus do not provide evidence that in these patients the motor system adjusts to the CST damage in a way that could be detected with fMRI and a simple motor task.

Many studies have previously demonstrated a relationship between fMRI brain activation and post-stroke outcome in patients with infarcts that spare M1. While the outcome of these studies varies to some extent, good recovery of motor performance has generally been associated with a preservation or restoration of activity in the ipsilesional hemisphere [11–14,42–45]. Sustained elevated task-related activity in the non-affected hemisphere has been associated with poor outcome [13,14,42–45]. Elevated recruitment of secondary and bilateral motor areas has been interpreted as a reflection of a compensatory strategy in patients who show poor recovery after stroke [46,47]. In accordance with previous research, longitudinal studies suggest that in the first weeks after stroke, movement of the affected hand is associated with overactivation within the bilateral sensorimotor network, and that this is more pronounced in patients with greater impairment [9,45,48].

The current results do not provide evidence for neuronal compensation beyond 6 months after stroke in patients with moderate (ARAT = < 57) to good (ARAT = 57) functional recovery. Even though multiple patients in the present study still showed significantly reduced speed of the upper paretic limb as reflected by reduced NHPT-scores, a more sensitive test of dexterity in our study, this did not induce detectable signs of neuronal plasticity. This is a negative finding which in principle places limits on the conclusions that can be drawn from this study. However, the comparatively large sample size in addition to the agreement with the observation that patients with good outcome at >6 months after stroke showed ‘normal’ activation compared to healthy controls [17], suggest that any differences in brain activity during this task in this particular experimental group are small at best.

Putatively, during the relatively simple fMRI task minimal motor output is sufficient for performance. However, with higher demands on the sensorimotor network (e.g. during the NHPT), the brain may switch to a new strategy that includes compensatory mechanisms. This was previously shown in patients where a higher exerted force induced higher activation in secondary motor areas [9,47]. Higher motor demands also might induce compensatory mechanisms in the musculoskeletal system by causing the intended action to be performed with different motor strategies [9,49]. Interpretation of differences in brain activity between patients and controls is clearly affected by the exact features of movement, in that different motor strategies are likely to engage the motor system in different ways which give little information about reorganization of brain function per se.

In our opinion, understanding the changes in activation in the affected and the non affected hemispheres after stroke found in literature, require a more fine distininction in measuring the quality of motor control after stroke. Future research should address how neural correlates of multi-joint movements change with increasing complexity in well recovered patients, while simultaneously assessing compensatory mechanisms in the musculoskeletal system using kinematic analyses [16,50,51].

Analysis of the data-glove data did not show evidence for overt mirror movements in patients or controls. However, the EMG-data showed isometric contractions correlating with the task in some patients as well as some controls. Since there was no difference in EMG score between patients and controls we do not expect this variable to affect our results. Interestingly, the occurrence of mirror isometric contractions was variable within patients. Mirror movements were often not present during both tasks. Therefore a check for mirror movements performed during the actual task is warranted. In addition, since some patients showed substantial isometric contractions of the extensor muscle of the arm, the mere observation of movement in the contralateral arm does not seem sufficient in assessing mirror contractions. While we did not observe evidence that during scanning isometric contractions had an effect on brain activity in this study, it is important to eliminate these confounding factors.

A flexion/extension task of the fingers was applied in the present study because it can be performed and monitored relatively easy in a scanner environment, and performance on this task during the first weeks after stroke is a good predictor of upper limb function at 3 and 6 months post stroke [6–8]. While our selected task was applicable in the scanner and clinically relevant, we did introduce a selection bias, by including only patients who showed some form of dexterity after stroke and in which the lesion might have spared some of the connections between the extensor muscle of the hand and M1 [6–8]. Although all of our included stroke patients showed clear signs of brain damage, and all reported impaired motor function after stroke indicating an insult to the sensorimotor system, there is thus still the possibility that in more poorly recovered patients there are more compensatory mechanisms in the arm during task. We do not believe however that the observation of more compensatory activation in more poorly recovered patients would be a straightforward sign of neuronal reorganization. These patients would most likely perform the task less well or in a different way using alternative preserved neuronal pathways [16]. Such a difference in performance could confound any evidence for neuronal plasticity. While the current patient group was only moderately impaired, we were able to perform an unbiased assessment of activity within the motor circuitry after stroke, and the absence of evidence for abnormalities in a relatively large sample may suggest that effects of true neuronal plasticity were small at best.

The current study contains several shortcomings that may have affected the sensitivity of our design. Most importantly, the patient population was quite heterogeneous regarding lesion location, which may have reduced statistical power by increasing variance within the patient group. While this issue is impossible to completely avert in stroke studies, we have attempted to lower its influence in a secondary analysis by including only patients of the largest subgroup (patients with lesions in the basal ganglia) and comparing them with control subjects. The overall pattern of results remained the same however. Secondly, for combining results of patients with left and right sided lesions, the fMRI results of some patients and controls were flipped across the interhemispheric fissure. While this procedure does not represent a confounding factor as it was done for both patients and controls, it can increase the variance within groups, and thus the sensitivity of the design. Finally, although the finger flexion/extension task we used is correlated to large muscle strength in the upper extremities, this relationship is incomplete. Although we believe that the choice for a finger motor task is optimal considering that large arm movements cannot be performed in the scanner without introduction of serious motion artifacts, it may have affected sensitivity in detecting abnormalities in some of the patients.

In conclusion, we did not find differences in brain activity between patients and controls, nor did we observe significant correlations with measures of outcome in patients. The absence of differences may suggest that functional reorganization in the sensorimotor network is not present in patients with good outcome. However, NHPT scores of patients indicated the motor system was compromised. While these patients show normal brain activation during simple finger extensions, this may not so with more challenging motor paradigms. With increasing task difficulty and increased taxing of the motor system, we may observe changes in motor system activation to overcome for the impairment. The same may happen when the current finger extension task would be performed by poorly recovered patients. It is however uncertain if observations of altered brain activity in the presence of differences in task performance could be regarded as true signs of neuronal plasticity.

## Supporting Information

S1 File Beta scores per ROI: Excel sheet containing the mean regressor coefficients for the different regions of interest for all patients and control subjects and all four conditions. (XLSX)

S2 File Contrast maps patients: Niftii files containing the contrast maps for the four different conditions vs. rest for all patients. (ZIP)

S3 File contrast maps controls: Niftii files containing the contrast maps for the four different conditions vs. rest for all control subjects. (ZIP)
